# Surveillance on the Status of Immune Cells after *Echinnococcus granulosus* Protoscoleces Infection in Balb/c Mice

**DOI:** 10.1371/journal.pone.0059746

**Published:** 2013-03-26

**Authors:** Wei Pan, He-Jun Zhou, Yu-Juan Shen, Ying Wang, Yu-Xin Xu, Yuan Hu, Yan-Yan Jiang, Zhong-Ying Yuan, Chidiebere E. Ugwu, Jian-Ping Cao

**Affiliations:** National Institute of Parasitic Diseases, Chinese Center for Disease Control and Prevention, Key Laboratory of Parasite and Vector Biology, Minstry of Health, World Health Organization Collaborating Centre for Malaria, Schistosomiasis and Filariasis, Shanghai, People's Republic of China; Fudan University, China

## Abstract

**Background:**

Cystic echinococcosis is a global parasitic disease caused by infection with *Echinococcus granulosus* larvae with potentially life-threatening complications in humans. To date, the status of the immune cells believed to be associated with the pathogenicity of *E*. *granulosus* infection has not been demonstrated clearly.

**Methodology/Principal Findings:**

In this study, we developed a multiplex flow cytometry assay to investigate the systemic immune status of innate and adaptive immunity at 30, 180, 360 days post-infection (dpi) in mice infected with *E*. *granulousus*. At 30 dpi, an increase in the number of CD11b^+^ and CD11c^+^ antigen-presenting cells (APCs) was observed. This was accompanied by the slight down-regulated expression of the co-stimulatory molecule MHC-II, indicating the impairment of APCs in early infection through the release of secretory-excretory products. In all infected groups, we observed a significant increase in innate immune cells, including APCs and GR-1^+^ cells, and a dramatic increase in the myeloid-derived suppressor cells (MDSC) expressing CD11b^+^/GR-1^+^. Moreover, the upregulation of the activated markers CD69, CD44, CD40L, and the downregulation of CD62L were observed in the CD4^+^ and CD8^+^ T cells following infection. Regulatory T cells expressing CD4^+^/CD25^+^/FoxP_3_^+^ increased significantly over the course of infection.

**Conclusions:**

Our findings demonstrate that the microenvironment in the peripheral immune system after *E*. *granulosus* infection changes in subtle but detectably ways, especially during the persistent period of infection. We found that T cells were activated following infection, but observed that the significant increase of immunosuppressive cells such as MDSC and Treg cells could inhibit T cell response to *E. granulosus* antigens. We suggest these cells may play a neglected but key role in the downregulation of the immune response in long-term parasitic infection. Understanding the basic functions and temporal interactions of these immunosuppressive cells will pave the way for new strategies of parasite vaccine design.

## Introduction

Cystic echinococcosis (CE, or hydatid disease) is a chronic endemic helminthic disease caused by infection with metacestodes (larval stages) of the tapeworm *Echinococcus granulosus*, and is one of the most widespread zoonotic diseases in humans in both developing and developed countries [1], [2], [3]. The hydatid cysts of *E*. *granulosus* develop as unilocular fluid-filled bladders within the internal organs (mainly the liver and lungs) of humans and other intermediate hosts. Clinical symptoms are mild during the early stage of infection when the cysts are gradually growing. At later stages, however, the parasite may physically damage tissues and organs and cause them to become dysfunctional. The spontaneous or provoked rupture of a parasitic cyst can be fatal. In addition, anaphylactic reactions, including urticaria, edema, respiratory symptoms, and anaphylactic shock, are well documented for CE [4]. Existing data suggests that the status of innate and adaptive immune cells changes following infection and that these changes are closely related to the pathogenicity of the disease in humans. However, the status of the innate and adaptive immune cells and their contributions to *E*. *granulosus* cyst progression remains poorly understood. Elucidating the characteristics of these immune cells will help develop new strategies for treatments.

Previous studies have focused mainly on the Th2 cell responses and cytokine profiles following *E*. *granulosus* infection, as these benefit parasite growth and development [5]. The results of these studies show that later stages of the infection are characterized by more dominant Th2 activity with elevated IL-4 and IL-10 levels and reduced IFN-gamma output in ConA- and antigen-stimulated splenocytes [5]. The circumparasitic leucocytes produce mainly IL-10 at five months post-infection [6]. In addition, the E/S products released by the parasites play key roles in immune evasion [7], [8]. *E*. *granulosus* escapes the host's immunosurveillance by interfering with monocyte differentiation and by modulating dendritic cells (DC) maturation [7]. This observation has been confirmed by the induction of apoptosis in DC and CD4^+^ CD25^+^ FoxP_3_^+^ T cells using cestode E/S-products [8], which suggests an important role for parasite persistence during chronic echinococcosis. However, systematic studies of host immune responses following *E*. *granulosus* infection are still lacking, and therefore, little information is available regarding the nature of these responses.

In this study, we investigated several immune cell populations involved in both innate and adaptive host immunity at various times post-infection in mice infected with *E*. *granulosus*. We aimed to (i) characterize host immune responses during the development of *E*. *granulosus* cysts in intermediate hosts and (ii) determine the interaction between the parasite and its host.

## Materials and Methods

### Ethics statement

This study was carried out in strict accordance with the recommendations in the Guide for the Care and Use of Laboratory Animals of the National Institute of Parasitic Diseases, Chinese Center for Disease Control and Prevention. The protocol was approved by the Laboratory Animal Welfare & Ethics Committee (LAWEC), National Institute of Parasitic Diseases, Chinese Center for Diseases Control and Prevention (Permit Number: IPD 2011-006). All surgery was performed under sodium pentobarbital anesthesia, and all efforts were made to minimize suffering.

### Mice, parasites and infection

Female Balb/c mice (aged 6 to 8 weeks) were purchased from the SLAC Laboratory (Shanghai, China) and were bred in the University facilities. The protoscoleces were obtained by aseptically puncturing the fertile bovine hydatid cysts according to protocols detailed in Baz et al. [9] and Andrew and John [10]. Briefly, the parasites were washed several times using phosphate buffered saline (PBS), pH 7.2, containing 1000 µg/mL penicillin and 1000 U/mL streptomycin (Invitrogen, Frederick, MD). The parasite vitality was determined by eosin exclusion [11]. Only parasite batches exhibiting over 90% vitality were used.

The Balb/c mice were inoculated intraperitoneally (in accordance with Araj et al. [12]) with 200 µL of a suspension containing 2000 live protoscoleces in PBS, whereas mice injected with 200 µL PBS were used as the controls. The mice were bred and housed normally until used for experimentation.

### Preparation of cell suspensions

Single cell suspensions were prepared from the murine spleens and peripheral blood after 30, 180, 360 day post-infection (dpi) with *E*. *granulosus* protoscoleces. First, the peripheral blood was collected, and the anticoagulant heparin sodium was added. Next, the mice were sacrificed and were soaked in 75% alcohol for 2 minutes. Finally, the sterile spleens were collected and placed in a clean plate. After two washes with PBS, the splenocytes were squeezed using the plunger of a 5 mL syringe and were passed through a 200 µm cell strainer (BD Biosciences) into 50 mL centrifuge tubes. The cell precipitations were washed using PBS and were centrifuged at 1000 rpm for 5 min. The cell precipitations, including splenocytes and peripheral blood cells, were hemolyzed in a solution containing 0.15 mol/L NH_4_Cl, 1 mmol/L KHCO_3_, 0.1 mmol/L EDTA and H_2_O (pH 7.2) for 5 min and were washed in PBS. The samples were spun at 300×g, incubated with anti-CD16/32 Fc-receptor blocking antibody for 10 min and washed in FACS buffer (2% BGS, 0.1% NaN_3_ in PBS) before surface staining. In this study, we omitted density gradient or enzymatic digestion steps to avoid selection bias, the loss of cells, and the modification of surface marker expression or cell function.

### Flow cytometry

The immune cells isolated from the mouse spleens or from the peripheral blood of mice were analyzed by flow cytometry. The spleen cells and the matched peripheral blood cells were harvested from infected and non-infected mice at different times post-infection. The aliquots of whole blood (100 µL), anticoagulated with EDTA, were incubated with the appropriate combinations of fluorescence-conjugated monoclonal antibodies. After lysing the erythrocytes with FACS lysing solution (BD Biosciences, Heidelberg, Germany), the cells were washed once in PBS (supplemented with 1% FCS and 0.2% NaN_3_) before assaying.

The cell suspensions were prepared in complete RPMI 1640. The non-specific binding sites were blocked for 30 min at 4°C using ice-cold PBS supplemented with 1% normal rat serum. After two washes, the cells were stained using the following anti-mouse antibodies: PerCP-CD8a (clone 53-6.7), FITC-CD4 (clone GK1.5), PerCP-Cy5.5–CD4 (clone GK1.5), APC-CD25 (clone PC61.5), PE-FoxP_3_ (clone MF-14), APC-IL-17R (clone PAJ-17R), FITC-CD11b (clone M1/70), PE-Ly-6G/Ly-6C (GR-1; clone R86-8C5), PE-CD40 (clone 3/23), Alexa Fluor 488-CD11c (clone N418), APC-I-A/I-E (MHC-II; clone M5/114.15.2), PE-CD3 (clone 17A2), and APC-CD45R/B220 (clone RA3-6B2). The antibodies were purchased from BD Biosciences (San Jose, CA). The anti-mouse CD69 PE (clone H1.2F3), CD154 APC (CD40L, clone MR1), CD44 APC (clone IM7), CD62L PerCP-Cy5.5 (clone MEL-14), was purchased from eBioscience (eBioscience, San Diego, CA). The incubation was performed for 30 min at 4°C. The CD4^+^ CD25^+^ FoxP3^+^ staining was performed according to the protocol provided in the Mouse Regulatory T cell staining Kit (eBioscience). The stained cells were analyzed on a FACS-Calibur SE flow cytometer (BD Biosciences). Prior to analysis, PI (propidiun iodide) was added to the cell suspensions to exclude the dead cells from the data acquisition. The data were analyzed using the FlowJo software package(Tree Star Inc., Stanford, USA).

### Proliferation assay

T cell proliferation was measured as previously described [13]. Briefly, splenic mononuclear cells from infected or control mice were stimulated with 3.5 µg/mL conA (Sigma-Aldrich, USA) for three days. After that, CCK-8 (10 µL/well) was added 4 h before harvesting. Data are the mean cpm ± SD of triplicate wells. Proliferation index was calculated as: cpm (conA stimulated group)/cpm (untreated group).

### Statistical analysis

Statistical analyses were performed using SPSS version 16.0 (Statistical Package for Social Sciences, Chicago, IL). Statistical significance was determined using Student's *t*-test and differences were considered statistically significant at P<0.05.

## Results

We found no significant differences in immune responses among the control groups of mice of different ages indicating that the spleen and peripheral blood content can be regarded as relatively stable under unchallenged conditions. Therefore, the untreated animals were used as baseline controls to calculate the P-values.

### Regulation of MHC-II expression in antigen presenting cells following infection

**Figure 1:**
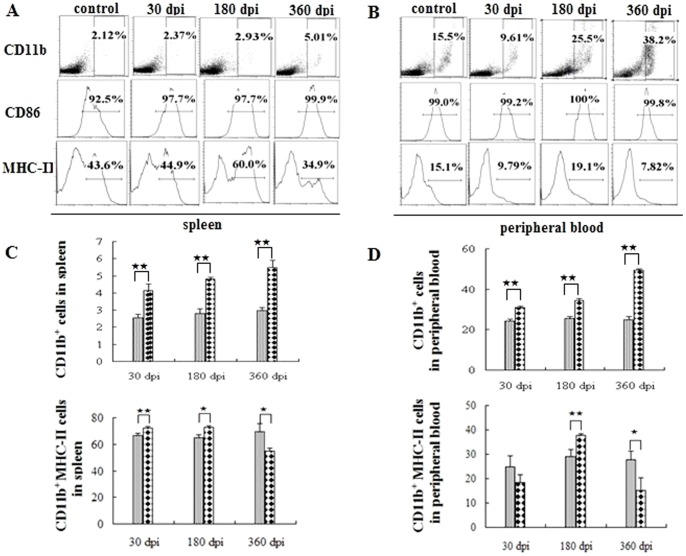
CD11b^+^ macrophages in the peripheral immune system post-infection. Proportion of CD11b^+^ cells in the spleen (A) and in the peripheral blood (B) in one of the six mice at 30, 180, 360 days post-infection (dpi). The age-matched control data are not shown in full because the levels are similar. Proportion of CD11b^+^ cells (C), CD11b^+^ MHC-II (D) in the peripheral immune system. The ash lattice pillars and the black lattice pillars represent the control and infected groups respectively. Plotted data are means ± SD of six mice. ★, P<0.05; ★★, P<0.01.

**Figure 2:**
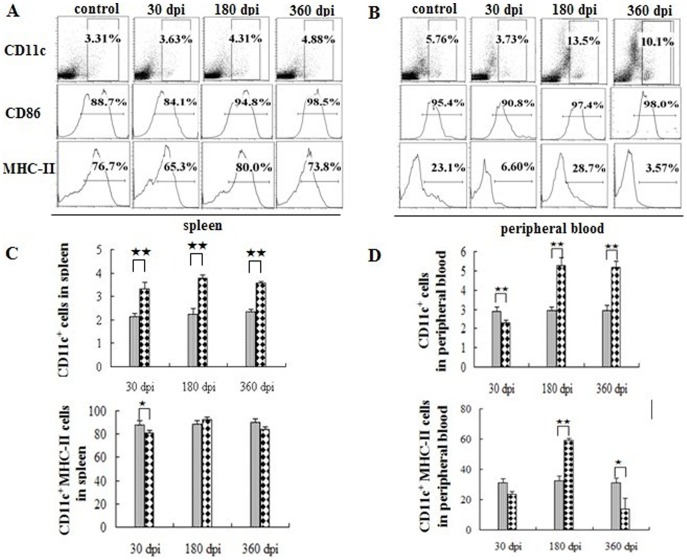
CD11c^+^ dentritic cells in the peripheral immune system post-infection. Proportion of CD11c^+^ cells in the spleen (A) and in the peripheral blood (B) in one of the six mice at 30, 180, 360 dpi. The age-matched control data are not shown in full. Proportion of CD11c^+^ cells (C) and CD11c^+^ MHC-II (D) in the peripheral immune system. The ash lattice pillars and the black lattice pillars represent the control and infected groups respectively. Plotted are means ± SD of six mice. ★, P<0.05; ★★, P<0.01.

Initially, we determined the antigen presenting cells (APCs) and the expression of the surface markers. The number of CD11b^+^ and CD11c^+^ cells in the peripheral immune system gradually increased post-infection (Fig. 1, Fig. 2). The CD86 expressed in these cells was also up-regulated following infection (Fig. 1A, 1B and Fig. 2A, 2B, middle). The expression of MHC-II in APCs showed a dramatic fluctuation in peripheral blood. In the spleen, more than 80% of the APCs cells expressed MHC-II over the entire course of the infection (Fig. 1C, 2C, lower). There was an up-regulation of MHC-II expression in the CD11b^+^ cells at 30 dpi and 180 dpi, however, the expression was apparently down-regulated at 360 dpi (Fig. 1C and 1D, left). Nevertheless, it exhibited the highest percentage of CD11b^+^ cells among the three groups. The fluctuation of the CD11c^+^ cells, which was similar to that seen for CD11b^+^ cells, exhibited an apparent increase post-infection (Fig. 2C and 2D). In the peripheral blood, MHC-II expression in APCs was apparently down-regulated at 30 dpi and 360 dpi, but there was a significant increase observed at 180 dpi (Fig. 1D and Fig. 2D).

### T cells were activated following infection

**Figure 3:**
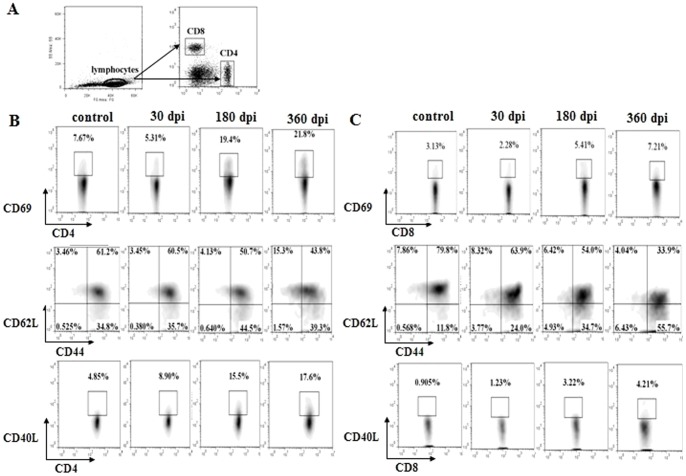
T cell activation in the spleen post-infection. (A) The gating strategies used for T cell activation analysis. (B) Proportion of CD4^+^/CD69^+^, CD4^+^/CD44^+^/CD62L^+^, CD4^+^/CD40L^+^ T cells in the spleen in one of the three mice at 30, 180, 360 dpi. (C) Proportion of CD8^+^/CD69^+^, CD8^+^/CD44^+^/CD62L^+^, CD8^+^/CD40L^+^ T cells in the spleen in one of the three mice at 30, 180, 360 dpi. The age-matched control data are not shown in full.

**Figure 4:**
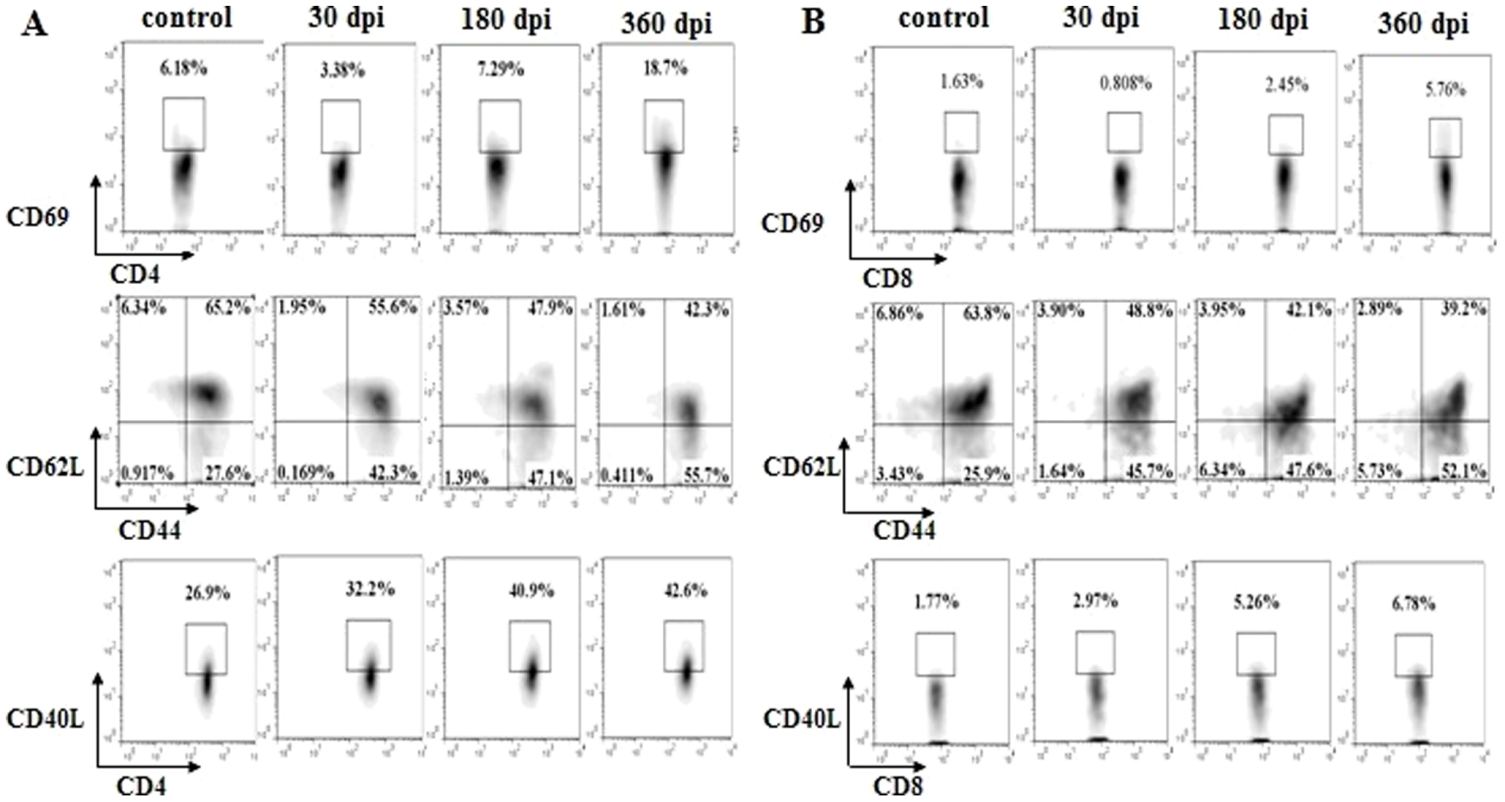
T cell activation in the peripheral blood post-infection. (A) Proportion of CD4^+^/CD69^+^, CD4^+^/CD44^+^/CD62L^+^, CD4^+^/CD40L^+^ T cells in the peripheral blood in one of the three mice at 30, 180, 360 dpi. (B) Proportion of CD8^+^/CD69^+^, CD8^+^/CD44^+^/CD62L^+^, CD8^+^/CD40L^+^ T cells in the peripheral blood in one of the three mice at 30, 180, 360 dpi. The age-matched control data are not shown in full. The gating strategies used for T cell activation analysis in the peripheral blood are the similar with Fig. 3A.

The number of T cells increased gradually following infection, although the infected spleens were not enlarged (data not shown). To determine whether the T cells were activated following infection, we stained the T lymphocytes for the expression of the activation markers CD69 [14], CD44/CD62L [15], CD40L [16]. The expression of CD69 in the CD4^+^ and CD8^+^ T cells was much lower at 30 dpi, however, a significant increase in expression levels was observed at 180 and 360 dpi (Fig. 3B, 3C and Fig. 4A, 4B, upper). The expression of CD44 and CD40L in the CD4^+^ and CD8^+^ T cells was gradually up-regulated post-infection, while the expression of CD62L was down-regulated (Fig. 3B, 3C, Fig. 4A, 4B, middle and lower). These data demonstrated that the T cells were activated following infection.

**Figure 5:**
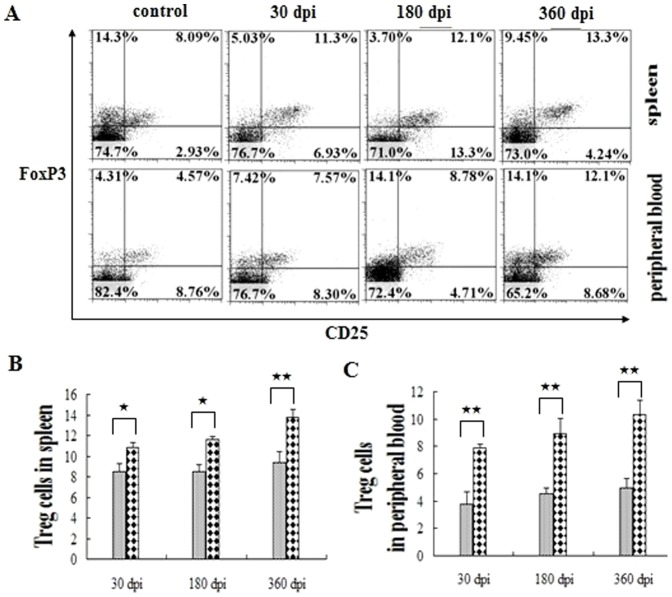
Regulatory T cells in the peripheral immune system post-infection. (A) Proportion of Treg cells in the peripheral immune system. The comparison of Treg cells in the spleen and peripheral blood in six mice are shown in (B) and (C), respectively. The ash lattice pillars and the black lattice pillars represent the control and infected groups respectively. The data represent the means ± SD of six mice. ★, P<0.05; ★★, P<0.01.

Meanwhile, we investigated the CD4^+^ CD25^+^ FoxP_3_^+^ T cells (regulatory T cells, Tregs) in the peripheral immune system (Fig. 5). The ratio of Tregs maintained a significantly higher level than that of the control group throughout the experiment, revealing their important role in suppressing the T cell activation post-infection (Fig. 5).

### Accumulation of myeloid-derived suppressor cells following infection

**Figure 6:**
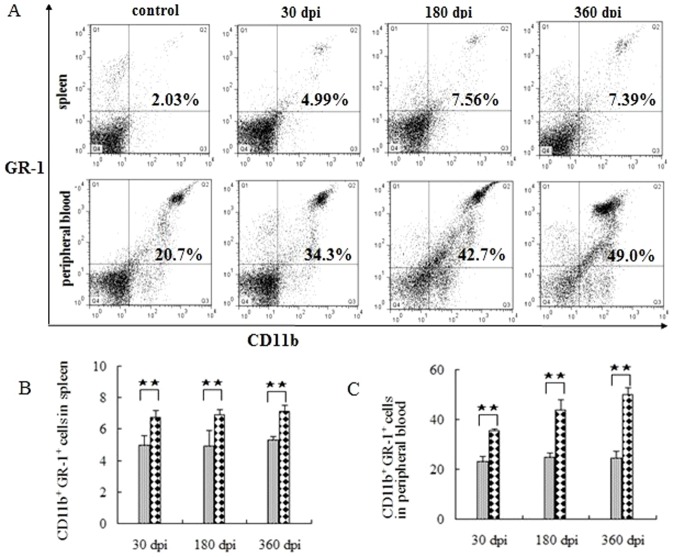
MDSC in the peripheral immune system post-infection. (A) Proportion of MDSC in one of the six mice at 30, 180, 360 dpi. The data showing the MDSC in the aged-matched controls are not shown in full because the levels are similar in every group. The comparison of MDSC in the spleen and peripheral blood are shown in (B) and (C), respectively. The data represent the means ± SD for six mice. ★, P<0.05; ★★, P<0.01.

In addition, the myeloid-derived suppressor cells (MDSC), defined as CD11b^+^ GR-1^+^ cells that contribute to tumor evasion, were investigated. The level of GR-1^+^ cells decreased at 30 dpi, but returned to normal levels at 180 dpi, and was significantly higher than in the control group (data not shown). The MDSC levels increased significantly following infection, especially in the peripheral blood (Fig. 6), demonstrating that MDSC plays an important role in the parasitic immune evasion through unknown mechanisms.

### The proliferation of T cells was significantly inhibited following infection

**Figure 7:**
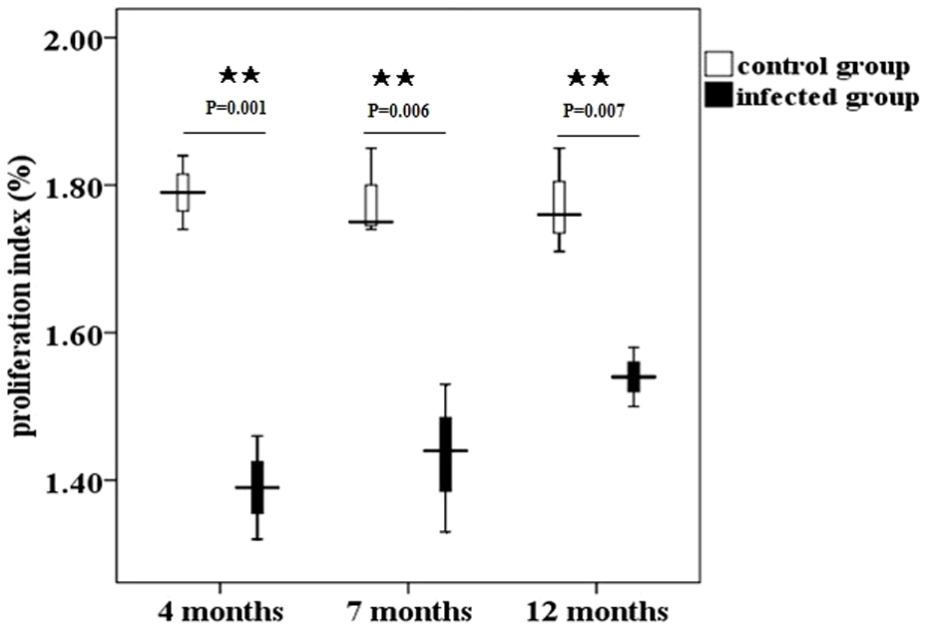
Splenocyte proliferation post-infection. Proliferation index of splenocytes from mice at four, seven, and twelve months post- infection measured by CCK-8 kit. The data represent the means ± SD for triplicate wells. ★, P<0.05; ★★, P<0.01.

To clarify the implications of the peripheral activation of splenocytes following infection, we performed functional stimulation tests. Proliferation assays using conA as stimulus revealed a significant decline in the proliferation index of splenic T-cells from infected spleens compared with control mice (Fig. 7). However, the ability of T cell proliferation was restored gradually post-infection (Fig. 7), suggesting T cell activation in the persistent period infection. In turn, this finding confirmed the vigorous inhibition of T cell response induced by immunosuppressive cells following infection.

## Discussion

Using multiplex flow cytometry, we described the key events in the cellular immune infiltration and activation following *E*. *granulosus* protoscoleces infection in Balb/c mice. We observed subtle changes in the microenvironments of both the spleen and the peripheral blood, including the infiltration and activation of innate APCs. In addition, we demonstrated for the first time the sequential activation of T lymphocytes and the accumulation of MDSC. Moreover, we observed a decrease in the proliferation index of splenic T cells stimulated with ConA, which reveals the vigorous suppressive effects induced by groups of suppressive cells in the peripheral system following infection.

In this study, we found that *E. granulosus* infection influenced the expression of MHC-II in the APCs and observed that the expression of MHC-II in the APCs declined at 30 dpi. There are several possible explanations for these observations. With the protoscoleces intrusion, the APCs were mobilized to present parasitic antigens, which could partly explain the APC depletion. In addition, E/S products released during the early period of infection can inhibit the activation of APCs and even cause apoptosis [8], which may mediate early immunosuppression. However, the immunosuppressive mechanisms have been established in the persistent period. At 180 dpi, the expression of MHC-II in the APCs was restored to normal levels. The fluctuation of MHC-II expression levels in the APCs demonstrates the complexity of the regulatory mechanisms that occur during the course of persistent infection. It is likely that the parasitic infection does not impair the expression of CD86 in the APCs because the up-regulation of CD86 in the APCs was observed in the infected groups.

The effect of anti-parasite immunity depends on the activation status of the T-lymphocytes. Naïve T cells are commonly characterized by the surface expression of L-selectin (CD62L) and the absence of the activation markers CD44, CD69 [15]. Naïve T cells can respond to novel pathogens that the immune system has not yet encountered. Recognition by a naïve T cell clone of its cognate antigen results in the initiation of an immune response. In turn, this results in the T cell acquiring an activated phenotype, which is indicated by the up-regulation of surface makers CD25^+^, CD44^+^, CD69^+^ and may further differentiate into a memory T cell [17]. Our results revealed that T cells were activated post-infection. Understanding why they could not eliminate the parasite infection through the host immune response requires further investigation. Han et al (2008) reported that CD69^+^ CD4^+^ CD25^−^ cells can suppress the host immune response to tumor cells [18]. The mechanism involves the inhibition of T cell activation through the TGF- beta pathway. Consistent with this observation, a high expression of TGF-beta is induced in *E*. *granulosus*-infected patients [19], [20]. It is possible that the CD69^+^ CD4^+^ CD25^−^ T cells play an immunosuppressive role in anti-parasite immunity. Meanwhile, consistent with other research work, increased levels of Treg cells have been found in CE patients [19] and in infected mice, revealing a role for Tregs in immune suppression in *E*. *granulosus* infection.

MDSC are a heterogeneous population of immature myeloid cells that consists of myeloid progenitors and precursors of DC, macrophages, granulocytes and myeloid cells [21]. Many studies have demonstrated that MDSC play key roles in the inhibition of anti-tumor immunity [22]–[24] through a variety of mechanisms including nutrient starvation [25], the generation of reactive oxygen and nitrogen species (ROS and RNOS) [26], [27], and the induction of regulatory T cells [28], [29]. There are few reports of MDSC in parasitic infections, such as *Schistosoma* [30]–[32], *Leishmania donaovani* [33], [34], and *Toxoplasma gondii* [35]–[37]. However, the MDSC-like cells in *Schistosoma mansoni* or *Leishmania donaovani* infections have not been well defined. Recent evidence in *Toxoplasma gondii* demonstrated that CD11b^+^ GR-1^+^ (CD11c^−^ Mac3 ^lo^) monocytic cells suppress the proliferation of ConA-stimulated lymphocytes [34], however, the mechanism of lung infiltration remains uncertain [35]. Therefore, the investigation of the importance of MDSC in parasitic immune evasion appears to have been neglected. In this study, the expression level of MDSC gradually increased throughout the course of the infection and was significantly higher during chronic infection. This high percentage of MDSC in the peripheral immune system demonstrates the importance of the cells in the immune response. However, to determine whether MDSC play key roles in immunosuppression during long-term infection with *E*. *granulosus* will require further investigation.

The peripheral microenvironment changed subtly following infection, although apparent pathological changes were not observed. However, under certain circumstances, the changes observed could pose a deadly threat to human. First, CE patients often manifest anaphylaxis [4]. The symptoms of anaphylaxis occur partly because of allergens in the cyst fluid, such as EgEF-1b/d [36], AE21 [37] and EgTeg [38]. In this study, E. *granulosus* infection did not induce apparent inflammation (data not shown). Instead, E. *granulosus* infection induced chronic inflammation including the infiltration of T lymphocytes, an increase numbers in APCs and GR-1+ granulocytes, and the accumulation of MDSC in the peripheral immune system. It is likely that vigorous immune responses could occur if the immune cells encounter parasitic antigens after a second exposure. In addition, neutrophils, which are known inducers of allergic reactions, represent a high percentage of the MDSC [39], [40], which contributes to a higher incidence of anaphylaxis in CE patients. Secondly, the immunosuppressive microenvironment post-infection often increases the chance of secondary pathogenic infections in the host. Therefore, disease progression is aggravated because the increased immunosuppressive cells can significantly decrease the protective immune response to a number of pathogens. Collectively, these changes contribute to pathogenetically relevant events.

## Conclusions

In this study, we developed a multiplex flow cytometry assay to investigate the status of innate and adaptive immunity at various times following *E*. *granulosus* infection in mice. Our data demonstrated subtle but detectable differences in the peripheral microenvironment post-infection and revealed that complex molecular networks were involved in the immune regulation. At 30 dpi, an increase in the number of APCs was observed accompanied by the down-regulated expression of the co-stimulatory molecule MHC-II, indicating the impairment of the APCs early in infection through the release of E/S products. Our study also demonstrates for the first time that MDSC, immunosuppressive cells exhibiting anti-tumor immunity increase significantly during persistent infection with *E*. *granulosus*. T cells were activated following infection, however, the significant increase in immunosuppressive cells such as MDSC and Treg cells could inhibit T cell response to *E. granulosus* antigens. Understanding the basic functions and temporal interactions of these immunosuppressive cells will pave the way for new strategies of parasite vaccine design.
